# Tracing social interactions in Pleistocene North America via 3D model analysis of stone tool asymmetry

**DOI:** 10.1371/journal.pone.0179933

**Published:** 2017-07-12

**Authors:** Sabrina B. Sholts, Joseph A. M. Gingerich, Stefan Schlager, Dennis J. Stanford, Sebastian K. T. S. Wärmländer

**Affiliations:** 1 Department of Anthropology, National Museum of Natural History, Smithsonian Institution, Washington, DC, United States of America; 2 Department of Sociology and Anthropology, Ohio University, Athens, United States of America; 3 Department of Anthropology, Medizinische Fakultät der Albert Ludwigs, University of Freiburg, Freiburg, Germany; 4 Department of Biochemistry and Biophysics, Stockholm University, Stockholm, Sweden; 5 UCLA/Getty Conservation Programme, Cotsen Institute of Archaeology, University of California Los Angeles, Los Angeles, California; Max Planck Institute for the Science of Human History, GERMANY

## Abstract

Stone tools, often the sole remnant of prehistoric hunter-gatherer behavior, are frequently used as evidence of ancient human mobility, resource use, and environmental adaptation. In North America, studies of morphological variation in projectile points have provided important insights into migration and interactions of human groups as early as 12–13 kya. Using new approaches to 3D imaging and morphometric analysis, we here quantify bifacial asymmetry among early North American projectile point styles to better understand changes in knapping technique and cultural transmission. Using a sample of 100 fluted bifaces of Clovis and post-Clovis styles in the eastern United States ca. 13,100–9,000 cal BP (i.e., Clovis, Debert-Vail, Bull Brook, Michaud-Neponset/Barnes, and Crowfield), we employed two different approaches for statistical shape analysis: our previously presented method for analysis of 2D flake scar contours, and a new approach for 3D surface analysis using spherical harmonics (SPHARM). Whereas bifacial asymmetry in point shape does not vary significantly across this stylistic sequence, our measure of asymmetric flake scar patterning shows temporal variation that may signify the beginning of regionalization among early New World colonists.

## Introduction

New approaches to morphological analysis of stone tools have produced a wealth of information about early human cultures [1–7]. Much of this research has focused on Clovis, the earliest well-documented cultural complex in North America ~ 13.5–12.8 kya [8–13]. Its principal diagnostic artifact, the Clovis point, is found throughout the continent with specific attributes of form (e.g., basal fluting and bifacial reduction) and technique (e.g., overshot flaking) that differ from subsequent projectile point styles in western and eastern North America [14]. Understanding how Clovis point shape variation relates to processes of cultural transmission, population movement, and subsistence adaptation can help illuminate changes in behavior and technology during the peopling of the Americas, the last major colonization event in human history.

Major questions about Clovis points are how this technology was acquired, transmitted, and modified across environments and over generations. Recent studies have produced different but not necessarily contradictory findings, possibly due to the effects of multiple learning strategies and evolutionary mechanisms on different point features [6]. In quantitative analyses of Clovis point size and shape, numerous samples and datasets have shown continent-wide uniformity or regional variation attributed to drift [15–17], whereas a recent geometric morphometrics (GM) analysis of 30 Clovis point assemblages across North America showed regional variation attributed to environmental adaptation [3]. Using shape analysis via outline-based Fourier analysis, which quantifies bifacial asymmetry in scar patterns originating from flake removal during the reduction process, we have previously demonstrated widespread production uniformity among Clovis points from across the United States [7].

One possible explanation for this apparent disagreement is that shape and flake scar patterning in Clovis points likely have different sources of variation. Shape is a deliberate and obvious feature with numerous functional properties, while flake scar patterning is the material signature of the tools and techniques used to create shape. We have previously shown that ancient Clovis flake scar patterns were significantly less variable than those made by a modern knapper, whose replicate points displayed no overall shape differences from the authentic ones from which they were copied [7]. Thus, we have proposed that Clovis flake scar patterns are the signature of a specific production technique passed between Clovis knappers by means of social learning and biased transmission [6, 7]. In a subsequent study of intraregional Clovis point shape variation, Eren and colleagues [4] found further support for this interpretation by comparing GM shape variables and a novel measure of flake scar “boldness”. Clovis points made from distinct stone outcrops showed significant differences in shape, yet the production technique appeared to be the same across the sample. These different patterns were explained by a model involving two tiers of social learning among Clovis knappers at stone outcrop hubs, where point shape would have diversified by processes of drift while knapping technique was maintained via conformist transmission [4].

In this study, we extend our investigations of point shapes and flake scar patterns to fluted point variation in the eastern United States, where the emergence of new fluted point styles following Clovis has been attributed to processes of regionalization and local environmental adaptation ~ 13–11.9 kya. Radiocarbon dates associated with several of these *a priori* point types or groupings provide a chronological framework to examine shifts in technology [5, 18]. Based on a phylogenetic analysis of a variety of shape characters, O’Brien and colleagues [19] suggested that a rapid proliferation of point forms after ~ 12,500 BP was the result of increased experimentation and individual learning with environmental and subsistence changes. Assuming that flake scar patterning and point shape are differently affected by environmental factors and adaptations, we here hypothesize that 1) when groups spread into new environments, the degree of flake scar asymmetry and the degree of overall point asymmetry will change differently. Second, assuming that flake scar symmetry reflects a Clovis knapping technique that was transmitted and maintained via social learning, we hypothesize that 2) during periods of decreased social learning/increased individual learning and experimentation, flake scar patterns in fluted point styles will be more asymmetric and variable.

**Fig 1:**
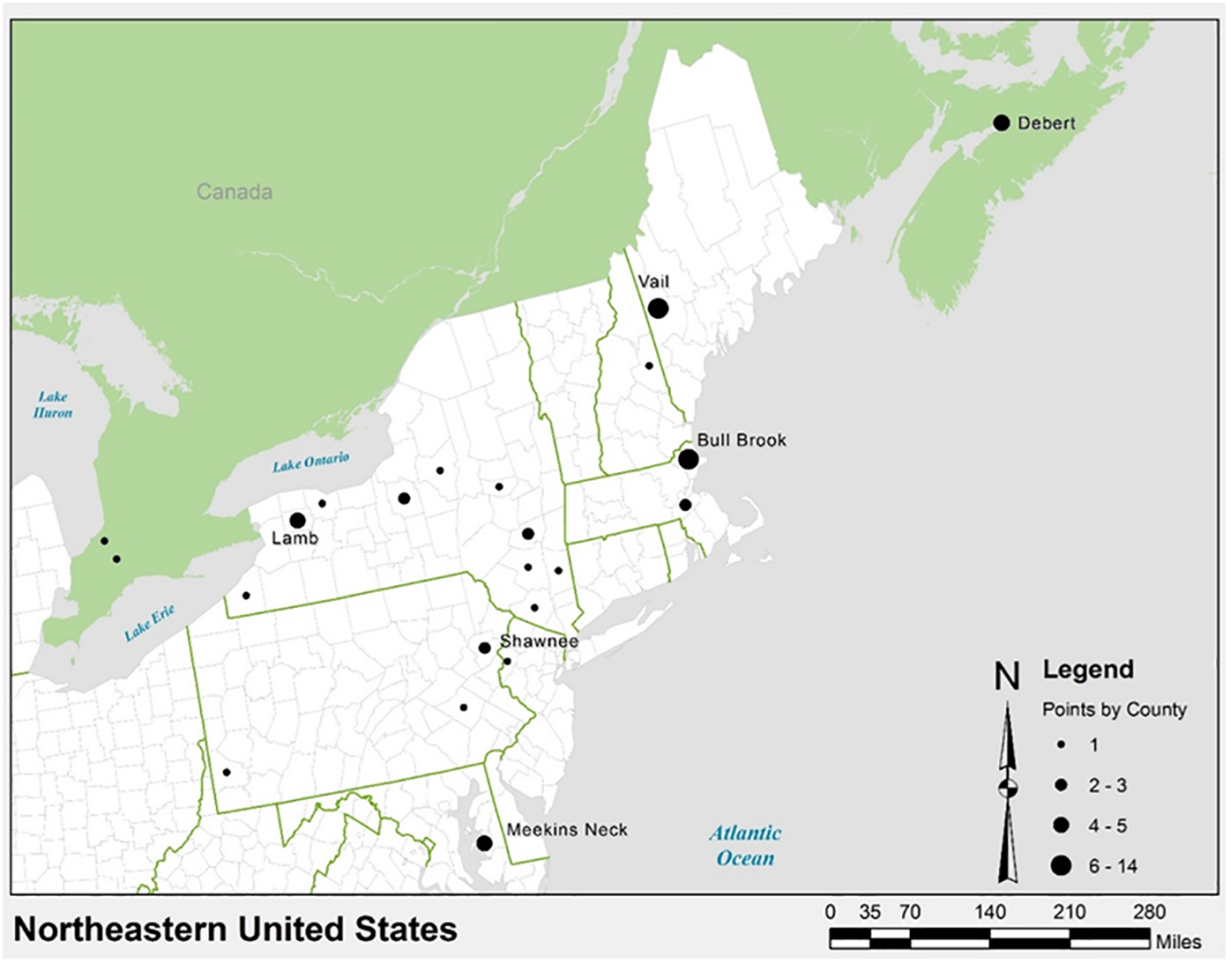
Geographic distribution of the eastern projectile points used in this study, with major sites listed. Map created by author JAMG using the ArcGIS 10.3.1 software.

**Fig 2:**
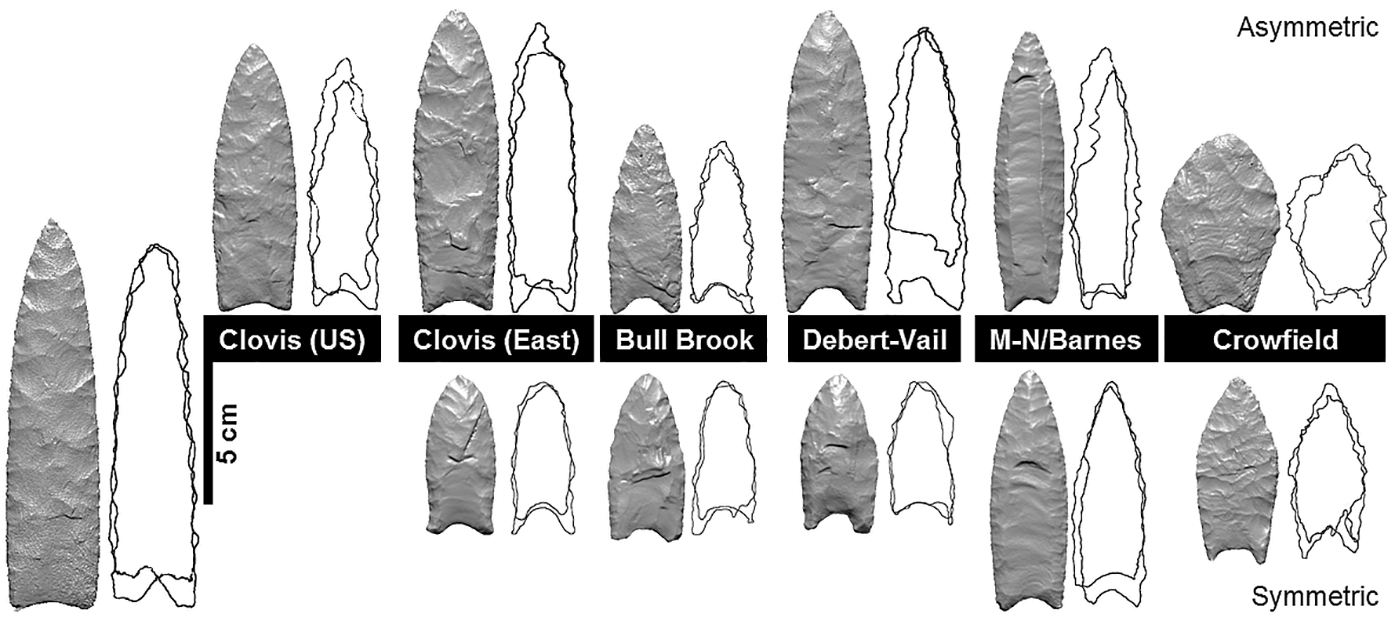
Three-dimensional models and their associated flake scar contours (superimposed flake scars from front and back faces) for projectile points of each style group. From left to right: Clovis US, Clovis East, Bull Brook, Debert-Vail, Michaud-Neponset Barnes, and Crowfield. Specimens displaying both high (top row) and low (bottom row) flake scar asymmetry are shown.

To test these hypotheses, we analyzed a sample of 100 fluted bifaces of Clovis and post-Clovis styles, i.e., Clovis, Debert-Vail, Bull Brook, Michaud-Neponset/Barnes (M-N/B), and Crowfield (Figs 1 and 2) using two different approaches for statistical shape analysis of bifacial asymmetry: our previously presented method for analysis of 2D flake scar contours [7], and a new approach for 3D surface analysis using spherical harmonics (SPHARM) [20]. Both approaches use digital 3D models to calculate degrees of asymmetry as displacement vectors reflecting the difference between front and back 2D contours or 3D surfaces. Thus, for each fluted biface, a single numerical value is calculated for flake scar asymmetry, and another single numerical value is calculated for topographic surface asymmetry. By applying both methods to the same sample, by showing a lack of correlation between asymmetry and physical dimensions and resharpening, and by relating between-group differences in biface asymmetry to differences in cultural behavior, we aim to better understand how different aspects of lithic asymmetry can inform us about the behavior of the individuals and groups who made these tools.

## Materials and methods

### Sample

This study included 100 fluted projectile points (S1 Table), which were assigned to one of the five style groups by authors JG and DS: Clovis, Debert-Vail, Bull Brook, Michaud-Neponset/Barnes (M/N-B), and Crowfield (Fig 2). Each point was assigned also to one of the following categories of reduction or retouching by authors JG and DS: 1) absent to moderate re-sharpening, 2) re-tipping, 3) re-basing, 4) re-tipping and re-basing, or 5) preform. Date ranges for each style group are given in Table 1 together with the morphological criteria used to differentiate them. Examples of points from each style group are shown in Fig 2. In the total sample, 67 points of all five styles are from the eastern United States (Fig 1, Table 1) and 33 Clovis points are from the central and western United States [7]. The latter set of projectile points was used in our previous study [7], and here this set is treated separately as a “Clovis US” style group, with the exception of five points from Maryland that were placed in the “Clovis East” group. To avoid ambiguity in the classification of points by style, a problem discussed at length by O’Brien and colleagues [19](page 105), our sample consists largely of projectile points from “type sites” such as Bull Brook and Debert-Vail, resulting in relatively small sample sizes for some style groups. The investigated points are currently located in collections at the Smithsonian National Museum of Natural History, New York State Museum, Canadian Civilizations Museum, Roberts S. Peabody Museum of Archaeology, Peabody Essex Museum, and Maine State Museum. A complete listing of the sample is presented in the S1 Table.

**Table 1:** Time periods and morphological definitions for the styles of projectile points analyzed in this study, adapted from [5]. Chronology estimated from discussions in [29] and [18]. Attribute list summarized from [29] and slightly revised for this study. Calibrated ages are presented as mean ages calculated from the CalPal online tool [30].

Style	Date range CalBP	Morphological features
Clovis	13,400–12,800	• Shallow basal concavity
		• Flute less than ½ the length of point
		• No developed mid-line
		• Fluting at or near center-line
Debert-Vail	12,700–12,630	• Deep basal concavity
		• Flute ½ to ⅓ the length of point
		• Lenticular cross-section
		• Generally parallel sides
		• Fluting below center-line
Bull Brook	12,630–12,160	• Moderate basal concavity
		• Multiple fluting common
		• Flute ½ to ⅓ the length of point
		• Slightly eared on occasion
		• Slightly divergent sides
		• Fluting below center-line (some preforms suggest use of indirect fluting)
Michaud-Neponset and Barnes (M/N-B)	12,160–11,900	• Moderate basal concavity
		• Fully fluted to flute >⅓ the length
		• Slight to moderate earing common
		• Divergent sides, presence of single underflute or Barnes finishing flake
		• Clearly developed midline
Crowfield	11,600–9,900?	• Pentagonal in shape
		• Medium in size
		• Very thin and flat
		• Narrow bases with shallow crescent-shaped concavity
		• Multiple fluting common, which occasionally includes overflaking of flutes

Regarding the temporal relationship between Clovis and other fluted point technologies, Clovis technology is the first continental-wide signature of people in North America, spanning from approximately 11,500 to 10,850 ^14^C BP (~13,400–12,800 calBP) [21–23]. Although Waters and Stafford have suggested a narrower time range for Clovis (ca. 11,050–10,800 ^14^C BP [12,950–12,770 CalBP]) [13], the vetting of Clovis radiocarbon ages to arrive at such an age range shows that Clovis technology synchronously appears across North America and shows no spatial patterning that would suggest a unique point of origin [13](page 1124). Radiocarbon dates, such as those in Florida (Sloth Hole), South Carolina (Topper), Virginia (Cactus Hill), Pennsylvania (Shawnee-Minisink), Paleo Crossing (Ohio) are equivalent to the majority of Clovis ages in the West [13, 18, 24, 25]. In the Northeast, Clovis age radiocarbon dates are rarely found above New York State [18]. Because of several environmental considerations these more northern areas of eastern North America are thought to have been colonized later than other regions [26–28]. How the later movement of people into the East may have affected the development of later fluted point technologies is unclear. Most researchers however agree that fluted points in the Northeast represent a continuation of the fluted point tradition that originated from the first widespread populations in North America. A more detailed discussion of the metric indices used to separate fluted point styles, together with a discussion of the individual radiocarbon dates associated with specific sites and or point styles, is provided in references [5, 18].

### 3D modeling

Following previously published protocols [7], digital surface 3D models of all fluted bifaces in the sample were created with a NextEngine desktop 3D laser scanner. Data capture settings were selected for maximum point density (400 DPI). The scan data were trimmed, aligned, and fused using tools in the ScanStudio CORE 1.7.3 software (NextEngine, Inc., 2006), in order to produce complete 3D models with single, continuous mesh triangle surfaces.

### Contour (2D) analysis

**Fig 3:**
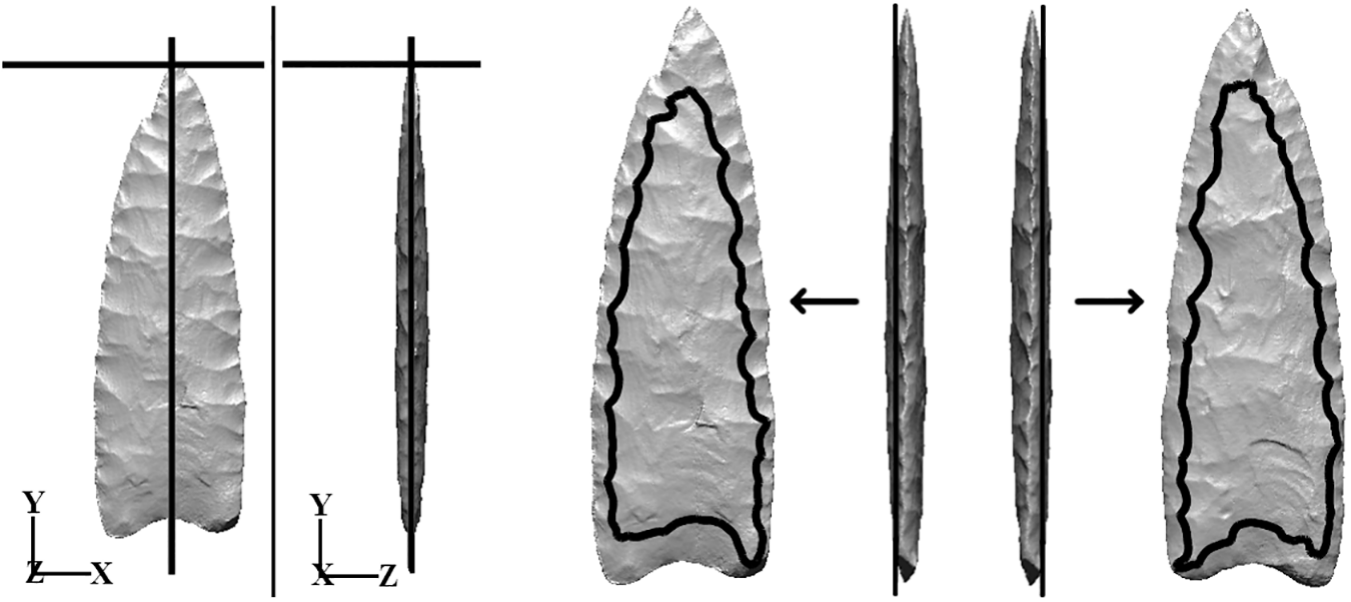
Flake scar contours from the front and back sides were obtained as isoheight contours created by intersecting the 3D surface model with two x−y planes, each offset a distance of ¼ total specimen thickness in the positive or negative z-direction. See Sholts et al. (2012) [7] for a full description of this process.

The finished 3D models were imported into the RapidWorks 64 2.3.3 program (NextEngine, Inc., 2008), where they were oriented and rotated according to a standard procedure (Fig 3). With the long-fluted face of the projectile point in front view, the geometry tools in the RapidWorks software were used to create a right-left symmetry (“mirror”) plane, along which the y-z plane of the coordinate system was defined. Next, a front-back symmetry plane was created, along which the x-y plane was defined. This yielded a coordinate system with the y-axis along the long axis of the object, the x-axis along the short axis of the object, and the z-axis perpendicular to the two faces (Fig 3). The origin of the coordinate system, i.e., (0, 0, 0), was set at the tip of each projectile point. From this standard position two auxiliary planes were created parallel to the x-y plane. The z-values for these two planes were defined as ¼ of the maximum thickness of the specimen, which was calculated as the difference in z-value between the most distant point in the positive z-direction and the most distant point in the negative z-direction. The intersections of the two auxiliary planes with the 3D model yielded two closed contours with uniform z-values (Fig 3; see also [7]). Along these isoheight contours, polylines with a fixed density of 3 points per mm were created. The x- and y-values of the data points were saved in standard text file format.

Elliptic Fourier (EF) coefficients to be used as shape descriptors were calculated using the R-package Momocs [31]. To account for differences in size, the length of the ellipse (i.e., the major axis) associated with the 1st EF harmonic was used to scale the original outlines. Subsequently, new EF coefficients (n = 32) were computed for the size-corrected contours. These EF descriptors were used to define a metric quantifying the amount of asymmetry between the front and back flake scar contours of a given projectile point. The basis for this metric is that after size standardization, two contours of identical shape have identical Fourier coefficients. Thus, the amount of asymmetry can be defined as the mathematical distance to the closest symmetric configuration of two contours. This metric is a modified multivariate extension of our previously published “symmetry distance” [7], defined by the following protocol:

To calculate the 2D asymmetry, we scaled the coefficients of all harmonics to unit standard deviation, in order to account for differences in variable scale. Next, we defined $x_{specimen_{i}}={\left(x_{1}^{\mathrm{front}},x_{2}^{\mathrm{front}},\ldots ,x_{n}^{front},x_{1}^{back},x_{2}^{back},\ldots ,x_{n}^{back}\right)}^{t}$ to be the shape vector for specimen number i, which contains the EF coefficients (for harmonics 1 to n) for both flake scar contours (i.e., front and back). In a linear space spanned by the coefficients, we then defined the orthonormal basis (*B*_*sym*_) of the subspace spanned by the symmetric shapes as:

$$B_{sym}=\left[\begin{array}{llll} 1/\sqrt{2} & 0 & \cdots & 0\\ 0 & 1/\sqrt{2} & & 0\\ \vdots & & \ddots & \vdots \\ 0 & \cdots & & 1/\sqrt{2}\\ 1/\sqrt{2} & 0 & \cdots & 0\\ 0 & 1/\sqrt{2} & & 0\\ \vdots & & \ddots & \vdots \\ 0 & \cdots & & 1/\sqrt{2} \end{array}\right]$$

The dimensions of this matrix are 2n *×* n, and $1/\sqrt{2}$ is a simple scale factor. In the space spanned by this basis, the *i-th* and the *(n+i)*-th vector entry (i < = n), holding to the i-th EF coefficient for the front and back contours, are identical. To obtain our metric for a specimen's overall asymmetry, we then simply calculated the distance from the shape vector to its orthogonal projection into that subspace as $asym=\|x_{specimen_{i}}-\left(B_{sym}B_{sym}^{t}\right)x_{specimen_{i}}\|$.

### Surface (3D) analysis

**Fig 4:**
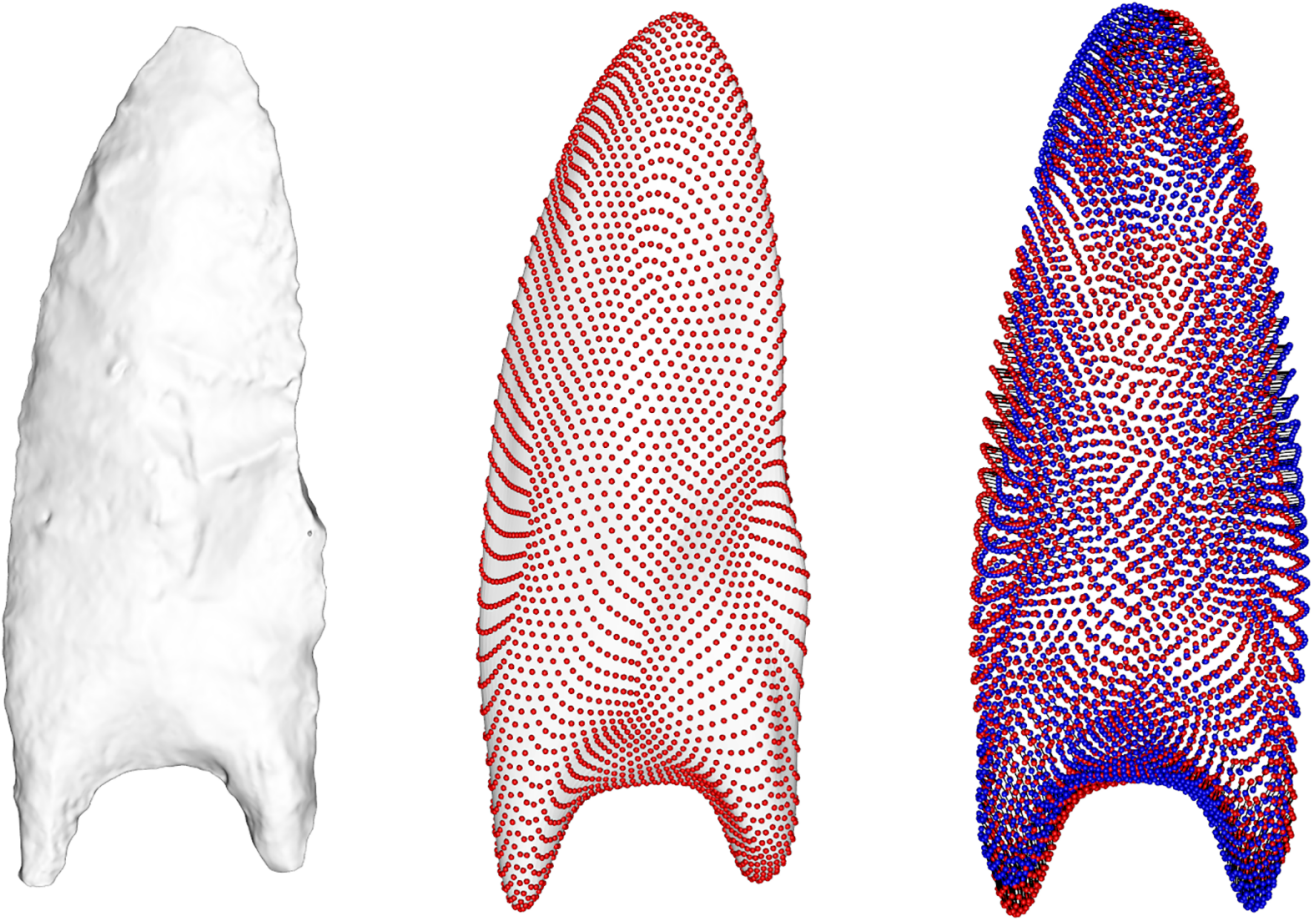
Left: Original surface mesh 3D model. Middle: Point distribution model (PDM) created using spherical harmonics (SPHARM) coefficients. Right: Overlaid front and back PDM surfaces used to calculate bifacial 3D surface asymmetry. The use of corresponding coordinates throughout the sample allowed a displacement field to be calculated as the difference between the front and back PDMs.

In order to employ the entire shape of the surfaces to quantify asymmetry difference between the two faces of a Clovis biface, we developed a protocol based on spherical harmonics analysis (SPHARM), an extension of elliptical Fourier analysis into the 3D domain. Based on the obtained harmonic coefficients, point distribution models (PDMs) were generated for each blade with coordinates corresponding throughout the sample. The details of the procedure are described below and illustrated in Fig 4. Calculations of SPHARM-PDMs was done using the command line tools available from https://github.com/NIRALUser/SPHARM-PDM. For the mathematical details of this procedure, see Nain et al. [20].

Mesh preprocessing was done with the R-packages mesheR, Rvcg [32], and RvtkStatismo. The basic approach was to parametrize the fluted point shapes as SPHARM-PDMs, then rotate the points 180° and apply an elastic deformation of the original version onto the rotated surface. This allows a displacement vector to be calculated between the perfectly symmetric average of the pre- and post-rotated 3D models and the actual 3D model shape. The length of this displacement vector constitutes a metric quantifying the shape difference between the two Clovis point faces. Our procedure included the following steps, starting with the 3D surface scans generated with the NextEngine laser scanner (see above). As the command line tools expect 3D-voxel data rather than surface meshes, we converted the mesh data using the following protocol:

First, all meshes were cleaned by removing isolated pieces, and then they were aligned along their principal axes. Subsequently all meshes were converted to 3D-images using the R-package RvtkStatismo. To ensure a similar resolution of the volume data relative to the points’ size, the image spacing was calculated by dividing the range along each dimension by 50. Based on these images, SPHARM-PDMs were calculated, resulting in surface meshes with 4002 vertices corresponding throughout the sample (and 8000 faces).

To generate difference vectors between these shapes and the rotated 3D models, each surface mesh was rotated 180° around its first principal axis and then rigidly aligned to the original version using a standard iterative closest point protocol [33]. To find the vertex correspondences between the original and the rotated 3D models, we applied a free-form deformation, regularized by a Gaussian smoothed displacement field [34] and additionally penalizing mesh distortions [35] following the procedure in Schlager and Metzger [36]. The shape differences associated with rotating were then calculated as the average vertex-deviation of the original version and the average of the original version and its deformation onto the rotated surface. To mitigate size differences, all shapes were scaled to unit centroid size. The resulting displacement vector is a direct extension of the asymmetry distance metric computed for the 2D flake contours (see above), with the main difference being that here the entire 3D geometry is used and analyzed, leading to a metric of overall difference.

### Statistical analysis

After computing 2D and 3D asymmetry measures for all specimens in the sample, between-group differences were analyzed using standard ANOVA and subsequent post-hoc pairwise comparisons, with p-values adjusted for multiple comparisons using the Holm-Bonferroni method [37]. Additionally, permutation tests (running 10,000 rounds) were used to control that the results were not biased from effects related to deviation from normality or small group size. F-tests for variance differences were performed on log-transformed asymmetry data, with p-values adjusted for multiple comparisons. As the asymmetry values are computed on a ratio scale, we also calculated coefficients of variation (CV; standard deviation/mean value) to facilitate between-group comparisons of variation.

To investigate whether the calculated 2D and 3D asymmetry measures were influenced by the physical dimensions of the specimens (i.e., length, width, thickness, surface area, and volume), robust gamma rank correlation coefficients and p-values based on permutation tests (10,000 rounds) were computed using the R-package rococo [38].

## Results

### Asymmetry and style groups

**Fig 5:**
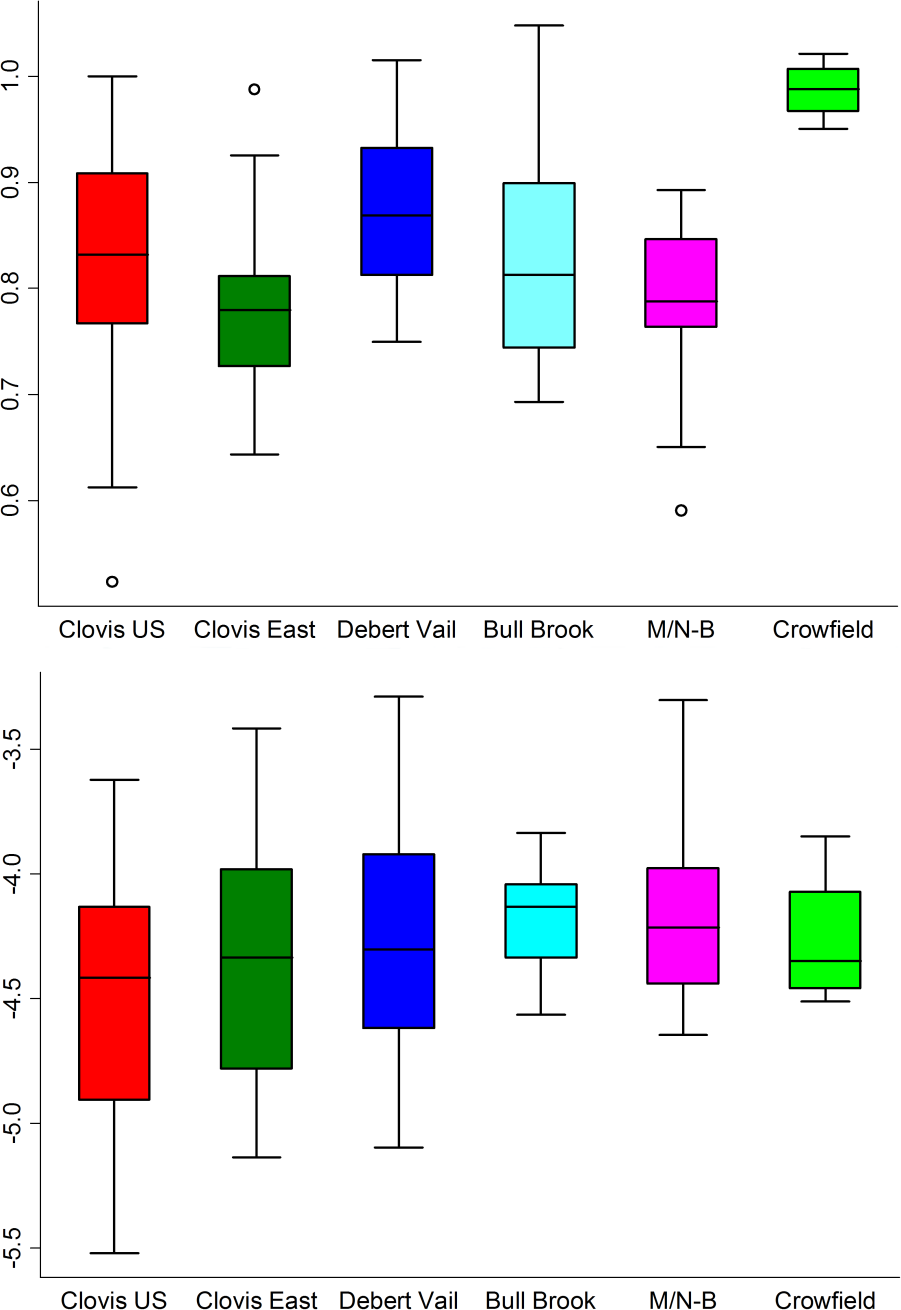
Distribution of log asymmetry values for 2D contours of flake scar patterns (top) and 3D biface surfaces (bottom) by style group.

The distributions of biface asymmetry values among different style groups, calculated from both 2D flake scar patterns and 3D surfaces, are shown in Table 2 and Fig 5.

**Table 2:** Asymmetry values for 2D contours of flake scar patterns (top) and 3D biface surfaces (bottom) by style group. SD = standard deviation, and CV = coefficient of variation, reported in %.

Style group	n	Mean	SD	Min	Max	Range	CV (%)
	**2D asymmetry**						
Clovis US	33	2.297	0.249	1.687	2.718	1.031	10.84
Clovis East	17	2.186	0.200	1.903	2.683	0.780	9.15
Debert-Vail	24	2.398	0.176	2.116	2.759	0.643	7.34
Bull Brook	12	2.303	0.252	2.000	2.851	0.851	10.94
M-N/B	10	2.183	0.196	1.806	2.442	0.636	8.98
Crowfield	4	2.683	0.078	2.587	2.777	0.190	2.91
	**3D asymmetry**						
Clovis US	33	0.012	0.006	0.004	0.027	0.023	49.53
Clovis East	17	0.015	0.008	0.006	0.033	0.027	51.33
Debert-Vail	24	0.016	0.008	0.006	0.037	0.031	50.15
Bull Brook	12	0.016	0.003	0.010	0.022	0.011	20.49
M-N/B	10	0.017	0.008	0.010	0.037	0.027	47.13
Crowfield	4	0.015	0.005	0.011	0.021	0.010	31.82

For 2D flake scar asymmetry, the group mean value for Crowfield, 2.68, is significantly higher than for the other groups, whose mean values range from 2.18 to 2.39. Debert-Vail is significantly higher than Clovis East and M-N/B (Tables 2 and 3). The F-tests show a significantly smaller asymmetry variance for Crowfield than for any other group (Tables 2 and 4). In line with these results, Debert-Vail and Crowfield display the lowest coefficients of variation (CV) values and Clovis US and Bull Brook the highest. The Crowfield results should, however, be interpreted with caution due to the very small group size.

**Table 3:** Pairwise p-values (corrected for type I error) from t-tests of differences in flake scar (2D contour) asymmetry between style groups.

Style group	Clovis US	Clovis East	Debert-Vail	Bull Brook	M-N/B
Clovis East	0.65	.	.	.	.
Debert-Vail	0.50	0.02	.	.	.
Bull Brook	1.00	0.86	0.86	.	.
M-N/B	0.86	1.00	0.07	0.86	.
Crowfield	0.01	0.00	0.08	0.02	0.00

**Table 4:** Results of F-tests for variance differences between log-transformed measures of flake scar (2D contour) asymmetry by style group.

Style group	Bull Brook	Clovis-US	Clovis-East	M-N/B	Debert
	**F-values**				
Clovis-US	1.02	.	.	.	.
Clovis-East	1.59	1.55	.	.	.
M-N/B	1.64	1.60	1.03	.	.
Debert	2.06	2.01	1.29	1.25	.
Crowfield	10.38	10.14	6.54	6.32	5.05
	***p*-values**				
Clovis-US	1.00	.	.	.	.
Clovis-East	1.00	1.00	.	.	.
M-N/B	1.00	1.00	1.00	.	.
Debert	0.63	0.62	1.00	1.00	.
Crowfield	0.01	0.01	0.04	0.15	0.10

For 3D surface asymmetry, there are no statistically significant group differences in mean or variance, even though the Crowfield and Bull Brook samples display somewhat lower standard deviations than the other groups (Tables 2, 5 and 6). In stark contrast to the 2D flake scar pattern data, Bull Brook displays by far the lowest 3D asymmetry CV value (20.49). These results clearly show that our 2D- and 3D- based shape analyses report on different aspects of biface asymmetry. The CV value for Crowfield (31.82) is also lower than for the other groups, which have values in the range of 47.1 to 51.3 (Table 2).

**Table 5:** Pairwise p-values (corrected for type I error) from t-tests of differences in surface (3D) asymmetry between style groups.

Style group	Clovis US	Clovis East	Debert-Vail	Bull Brook	M-N/B
Clovis East	1.00	.	.	.	.
Debert-Vail	1.00	1.00	.	.	.
Bull Brook	0.42	1.00	1.00	.	.
M-N/B	1.00	1.00	1.00	1.00	.
Crowfield	1.00	1.00	1.00	1.00	1.00

**Table 6:** Results of F-tests for variance differences between log-transformed measures of surface (3D) asymmetry by style group.

Style group	Clovis-US	Clovis-East	Debert	M-N/B	Crowfield
	**F-values**				
Clovis-East	1.08				
Debert	1.23	1.14			
M-N/B	1.72	1.58	1.39		
Crowfield	3.22	2.97	2.61	1.87	
Bull Brook	5.86	5.41	4.76	3.41	1.82
	***p*-values**				
Clovis-East	1	.	.	.	.
Debert	1	1	.	.	.
M-N/B	1	1	1	.	.
Crowfield	0.11	0.11	0.11	1	.
Bull Brook	0.10	0.10	0.11	0.60	1

### Asymmetry, physical dimensions, and retouching

No strong correlations were found between 2D flake scar asymmetry and the physical dimensions of the projectile points (Table 7)—only a weak correlation with maximum point width (r = -0.22). For 3D surface surface asymmetry, stronger correlations (Table 7) were observed for point length (r = 0.50), volume (r = -0.44), and surface area (r = -0.45), while weaker correlations were observed for width (r = -0.22) and thickness (r = -0.20).

**Table 7:** P-values and robust gamma rank correlation coefficients between physical dimensions of the projectile points in the sample and log asymmetry values for respectively 2D contours of flake scar patterns (left) and 3D biface surfaces (right).

Physical dimensions	log asymmetry			
	2D		3D	
	r	*p*	r	*p*
Length	-0.048	0.52	-0.51	0.0000
Surface area	-0.020	0.79	-0.45	0.0000
Thickness	-0.14	0.057	-0.20	0.0055
Volume	-0.087	0.23	-0.43	0.0000
Width	-0.22	0.0026	-0.22	0.0039

Descriptive statistics of asymmetry for each retouching category are reported in the S2 Table. Although the considerable differences in the number of specimens in the different categories prevent any meaningful statistical comparisons between their distributions, it is clear that there are no systematic patterns of covariation between projectile point asymmetry and degree of resharpening.

## Discussion

The slow change over time in fluted point technologies in eastern North America provides an ideal case study to examine technological variation and change during the Paleoindian period. Unlike in the western North America, where a relatively abrupt shift in fluted point technology occurs with the appearance of Folsom and the disappearance of Clovis around 10,900–10,800 ^14^C BP [39], the fluted point tradition in the eastern North America consists of a number of different ‘types’ or ‘styles’ that persist for almost 1,000 radiocarbon years [18]. Because the transition to the full-fluted form (i.e., the Barnes/Michaud-Neponset style) occurs much later in the East (10,200–10,300 ^14^C BP), morphological analyses have the potential to track changes in fluted point technology across the region as groups of people moved into new parts of the continent, or developed new styles and reduction techniques over time or within territories. The particular morphological aspect studied in this work is bifacial asymmetry, which we consider to be informative about knapping technique and skill.

Asymmetry measurements have long been used in anthropological research, often motivated by the inherent physical symmetry found in humans and other bilaterian species [40]. In such organisms, asymmetries are seen as departures from an ideal developmental program caused by environmental stress and/or genetic mutation [41]. Fluctuating asymmetry, i.e., normally distributed differences about a mean of zero difference, has served as the basis of numerous measures of developmental “noise” or instability [42]. Measurements of fluctuating asymmetry have shown significant correlations with perceived physical attractiveness in humans [43], as well as with mating success in many other species [44]. Cognitive studies indicate a preference for bilateral symmetry in the human visual system and support the existence of pre-attentive processes of symmetry detection [45]. This preference might explain why symmetry is often found in human-made objects that lack a genetic rationale for it. Another explanation is that for objects made from stone and most other materials, a symmetric design will distribute loads and stresses evenly, thereby reducing the probability that the material will break, crack, or otherwise fail. In arrowheads or spear points, an asymmetric weight distribution also may induce undesired wobbling in flight [46, 47]. The reasons for symmetry in objects are however less understood than those for symmetry in biological organisms. Thus, although our previous research suggests that bifacial symmetry in flake scar patterns is a unifying trait of Clovis-style knapping techniques and projectile point forms [7], the importance and influence of such symmetry in post-Clovis fluted point styles is unclear. To some extent this problem is analogous to the challenges faced by organismal biologists in distinguishing forms of bilateral asymmetry that reflect normal development, i.e., fluctuating and directional asymmetry and anti-symmetry [48], from the symmetry-breaking effects of different genetic and environmental factors [49].

### Hypothesis 1: 2D versus 3D bifacial asymmetry

Bifacial flake scar asymmetry was calculated according to a protocol we previously developed [5, 7], which involves 2D contours being extracted from 3D models and evaluated with elliptic Fourier analysis, a mathematical tool developed in 1982 [50]. The asymmetry of the biface surfaces was computed by analyzing the digital 3D models with spherical harmonics, a mathematical extension of elliptic Fourier analysis into 3D space developed during the last decade [20]. Thus, we here continue our efforts to develop new ways to investigate archaeological material using 3D technology [51–58]. With new mathematical tools available for analyzing 3D surfaces, it becomes natural to ask what kind of information can be obtained from respectively 2D contours and 3D surfaces [59]. For the fluted bifaces studied here, we argue that the two report on different aspects of asymmetry, as flake scars are unintentional by-products of tool manufacture, while the overall surface shape reflects the original intentional design together with gradual changes effected during the tools use life (e.g., use-wear and re-sharpening).

The correlation coefficients reported in Table 7 show that there is no strong correlation between flake scar asymmetry or surface asymmetry and the physical dimensions of the fluted points. The S2 Table furthermore indicates no co-variation between symmetry and biface resharpening evaluated by retouch category. These results are consistent with previous evidence that resharpening is not a significant source of population-level variation in the overall form of Clovis points in the Midwest (2). Between groups, bifacial asymmetry shows more variation in flake scar patterns than in surface morphology. These findings may not be surprising, as the overall design pattern for these tools is bifacially symmetrical, and as the asymmetries in flake scar patterns only contribute a small part to the amount of overall surface asymmetry. Thus, both 3D surface asymmetry and 2D flake scar asymmetry appear to be generally independent of the recorded biface dimensions and attributes. Both asymmetry metrics should therefore be useful as independent reporters of aspects of tool shape and symmetry.

As a case in point, our results show that Bull Brook is the group with both the largest CV variation for 2D flake scar asymmetry and the smallest CV variation for 3D surface asymmetry (Table 2). While these findings support our hypothesis that these characteristics differ in variation as groups spread across and adapted to new environments, the specific reasons behind these differences are unclear. In the case of Bull Brook, it appears that knappers used more variable techniques of manufacture but aimed to achieve the same overall point design, which may be the result of a stylistic preference, functional requirements, or simply the absence of selective pressure(s) for modification. As no other groups show a similar inverse relationship, this issue is an interesting topic for further investigation.

### Hypothesis 2: Bifacial asymmetry and lithic technology

Our calculated coefficients of variation (Table 2), a commonly used indicator of change in transmission processes [60–62], show that the flake scar asymmetry is even lower and less variable in the Clovis East group than in the larger Clovis US group (i.e., in our earlier studied sample [7]). These results are in line with recent findings of consistency in Clovis knapping techniques within a small geographic region of the eastern United States (4). Although Eren and colleagues (4) quantified unifacial rather than bifacial flake scar patterning, i.e., outer-to-inner flake scar count ratio, their findings together with our current results suggest that both attributes are tied to the biased transmission of Clovis point manufacture within and between regional groups. After the Clovis era, fluted projectile points in the eastern United States display a range of shape differences such as deeper basal concavities (e.g., Debert-Vail), divergent sides and eared bases (e.g., Bull Brook and M-N/B), clearly developed midlines (e.g., M-N/B), and pentagonal silhouettes (e.g., Crowfield) (Fig 2; Table 1). Except for Bull Brook, all of these later point styles show less within-group flake scar asymmetry variation than the Clovis U.S. and Clovis East samples (Table 2). The most straightforward explanation for this observation is the emergence of regional standardization, as the later styles did not become as geographically widespread as Clovis.

Bull Brook, on the other hand, displays higher within-group variation in flake scar asymmetry (CV = 10.94; Table 2) than any other group. Together with the very low within-group variation in 3D surface asymmetry (CV = 20.49; Table 2), this finding supports the idea of increased experimentation and individual learning around 12,500 BP previously proposed by O’Brien and colleagues [19]. As discussed by Eerkens and Lipo [61], simple copying errors can account for small changes in variation and drift-like processes of increasing variation over time, whereas processes of invention, discovery, and/or innovation are expected to generate variation on a scale much higher relative to copying error. Inadvertent copying errors in stone tool manufacture have previously been compared to genetic mutations that result in random (selectively neutral) variation, in contrast to intentional cognitive mechanisms that result in directional variation of much greater magnitude [63]. When applied to Clovis projectile points, a model of biased accumulation of copying errors has successfully predicted patterns of size variation across North America, supporting the hypothesis that Clovis point technology was highly stable and subject to biased transmission through broad social networks throughout the continent [16]. Those findings are consistent with our previous results showing that independently developed knapping techniques did not produce the same degree of bifacial flake symmetry as archaeological specimens, although the latter were visually indistinguishable from the replicated bifaces [7]. Thus, our results here support the hypothesis that flake scar asymmetry increases with (ostensible) decreases in social learning, most likely compounded by drift-like processes.

With the M-N/B style, Paleoindian groups began making their points with full flutes, which followed a carefully constructed central ridge that was created during the reduction process. Thus, this style reflects a significant technological change in fluted point reduction techniques in the eastern United States. The more symmetrical and less variable flake scar patterns we observe in the M-N/B group, compared to the preceding Bull Brook style (Table 2), suggest a return to the proposed Clovis-type of knapping behavior, in which reduction techniques were regulated by modes of social learning. Such consistency in manufacturing techniques has been associated with the introduction of new technology, as less variation occurs during a period of careful copying or adoption of behaviors, i.e., prestige-biased or conformist transmission [60, 61]. Another possible reason for decreased experimentation could be related to changes in lithic procurement patterns brought about by changes in mobility. In the Northeast, several archaeological sites show evidence of the final stages of biface production far from lithic sources, such as the Colebrook site in New Hampshire, where 73 channel flakes of munsungun chert were found, with no broken preforms, more than 300 km from the quarry site [64, 65]. In an effort to conserve lithic raw material by reducing failures in tool manufacture, the most skilled knappers may have been the primary producers of tools at these site and thus produced more symmetric points. Likewise, if increased mobility during the M-N/B period resulted in groups being farther from quarry locations, the more frequent use of specialists to help conserve raw materials would have led to fewer knappers with a limited reduction repertoire (see also [66]). The smaller territories exploited by M-N/B groups compared to early groups [63] also may have increased social interactions and biased modes of cultural transmission.

While the lower bifacial asymmetry values associated with M-N/B may indicate a return to a biased mode of cultural transmission similar to that for Clovis, the consistently high bifacial asymmetry in the Crowfield sample (Table 2) is compatible with the drastic change in overall shape. Although the small sample size for Crowfield warrants caution with any interpretation, developing the new features of this style must have involved a fair amount of experimentation regarding function and manufacture.

## Conclusions

This study builds on a recent body of research employing novel methods for quantifying variation in biface morphology and interpreting such variation in the context of social interaction, environmental adaptation, and New World colonization [4–7, 15]. We show that biface asymmetry can be quantified from two different approaches to shape analysis reporting on different aspects of asymmetry, i.e., 2D flake scar contours analyzed via elliptic Fourier analysis, and topological 3D surfaces analyzed via spherical harmonics calculations. Supporting previous findings [4, 5], flake scar asymmetry computed from 2D contours is shown to be independent from overall point shape and 3D surface asymmetry, as well as from resharpening and size variables. Flake scar asymmetry also appears to be more responsive to changes in manufacturing technology compared to 3D surface asymmetry, and should continue to be explored as a unique and valuable source of biface variation. Although social and environmental adaptations cannot easily be divorced from one another, our interpretation of flake scar patterns based on evidence of cultural transmission supports the traditional view of Clovis as a wide-spread cultural and technological tradition that was maintained over an environmentally diverse continent, and which was succeeded by more regional styles displaying higher levels of standardization.

## Supporting information

S1 Table Sample data. (TXT)

S2 Table Contour-based (2D) and surface-based (3D) asymmetry values by retouching category. (PDF)
