# Supplementary material for: Tracing social interactions in Pleistocene North America via 3D model analysis of stone tool asymmetry
Source: PLoS One. 2017 Jul 12;12(7):e0179933. doi: 10.1371/journal.pone.0179933 (PMC5507483; doi:10.1371/journal.pone.0179933)
Supplement: S2 Table — (PDF) [file pone.0179933.s002.pdf]

Retouching category	n	Mean	SD	Min	Max	Range
			**2D asymmetry**			
Resharpened (1)	77	2.28	0.23	1.69	2.78	0.86
Rebased (2)	10	2.47	0.23	2.06	2.85	0.79
Retipped (3)	5	2.41	0.20	2.22	2.68	0.47
Rebased and retipped (4)	5	2.31	0.22	2.11	2.61	0.51
Preform (5)	3	2.19	0.55	1.81	2.58	0.77
			**3D asymmetry**			
Resharpened (1)	77	0.01	0.01	0.00	0.03	0.03
Rebased (2)	10	0.02	0.00	0.01	0.02	0.01
Retipped (3)	5	0.02	0.01	0.01	0.04	0.02
Rebased and retipped (4)	5	0.02	0.01	0.01	0.03	0.02
Preform (5)	3	0.02	0.02	0.01	0.04	0.02

Supporting information Table S2. Contour-based (2D) and surface-based (3D) asymmetry values by retouching category.
